# Diagnostic accuracy of reflectance confocal microscopy for acral lentiginous melanoma

**DOI:** 10.1016/j.jdcr.2025.11.007

**Published:** 2025-11-13

**Authors:** Una Milovanovic, Adrianne Pan, Jennifer Laborada, Lucy Lam, Patrick Ottuso, Sairekha Ravichandran, Lilia Correa-Selm

**Affiliations:** a Nova Southeastern University, Dr. Kiran C. Patel College of Allopathic Medicine, Fort Lauderdale, Florida; b Department of Dermatology and Cutaneous Surgery, University of South Florida Morsani, Tampa, Florida; c Advanced Dermatology and Cosmetic Surgery, Vero Beach, Florida; d Cutaneous Oncology Department, Moffitt Cancer Center, Tampa, Florida

**Keywords:** acral lentiginous melanoma, confocal microscopy, diagnostic accuracy, histology correlation, noninvasive imaging, pigmented acral lesion, targeted biopsy

## Clinical presentation

**Fig 1:**
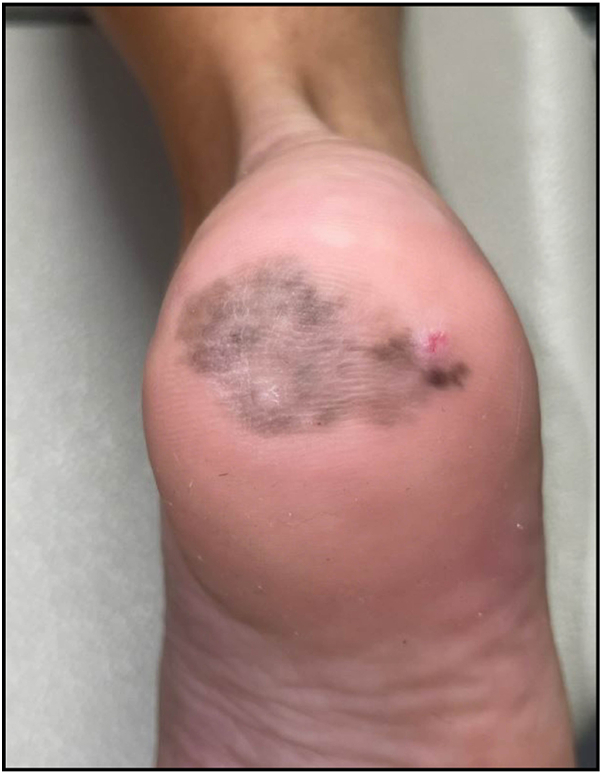
A 3.5 × 2.3 cm *gray-brown macule* with irregular borders is seen on the plantar aspect of the right heel.

A 51-year-old Caucasian woman with a history of melanoma presented with a brown patch on the right heel. The patient described its onset 10 years ago and reported that the lesion had been changing in size and color in the recent years (Fig 1). Following an initial shave biopsy that was inconclusive, a punch biopsy was subsequently performed. The results suggested lentigo with negative staining for Preferentially Expressed Antigen in Melanoma. An independent dermatopathology review was obtained, which supported the initial findings. However, due to the small sample size, a repeat biopsy was recommended to achieve a more accurate representation of the larger lesion. Given the persistent diagnostic uncertainty and the lesion’s atypical and evolving characteristics, the patient was referred to for reflectance confocal microscopy (RCM) evaluation to have a better assessment of the lesion.

## Confocal microscopy appearance

**Fig 2:**
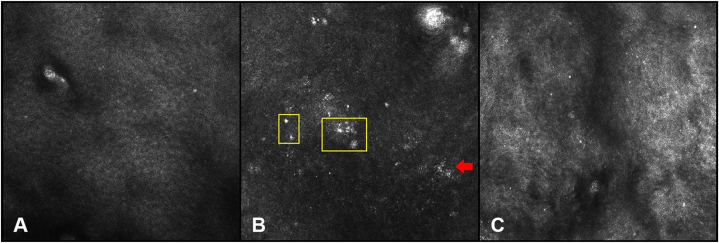
**A,** Stratum spinosum of the epidermis showing a normal honeycomb pattern formed by keratinocytes. **B,** Nucleated melanocytes (*yellow square*) and multiple lymphocytes representing inflammation (*red arrow*). **C,** Upper dermis represented by collagen fibers.

RCM of the lesion was obtained using a VivaScope 1500 (Caliber Imaging and Diagnostics). The real-time, high-resolution imaging revealed focal collections of atypical melanocytes with bright, pleomorphic nuclei at the dermal–epidermal junction, findings consistent with melanoma (Fig 2).

## Histological diagnosis

**Fig 3:**
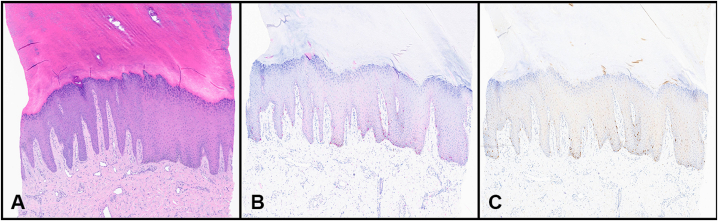
**A,** Hematoxylin-eosin–stained tissue section from the right heel reveals increased numbers of single junctional melanocytes, some with nuclear atypia. **B,** SOX10 immunohistochemical stain with appropriate positive control highlights the distribution of melanocytes within the epidermis and highlights the variable nuclear size of the atypical melanocytes. **C,** PRAME immunohistochemical stain with appropriate positive control is strongly positive among the atypical junctional melanocytes. *PRAME*, Preferentially Expressed Antigen in Melanoma.

Following the concerning RCM findings, additional, broader biopsies were performed targeting areas of the lesion that showed atypia under RCM. Histopathologic examination of these samples confirmed the diagnosis of acral lentiginous melanoma (ALM) in situ (Fig 3).

## Key message

Histopathologic diagnosis of early-stage ALM remains challenging, as cytologic atypia is minimal and architectural disorder subtle.^1^ Evaluation is further complicated in large or clinically heterogeneous acral lesions, where difficulty in obtaining a representative tissue sample increases the risk of diagnostic delay.^2^ In this context, RCM provides a noninvasive, high-resolution imaging modality that enables real-time, near-histologic evaluation of the entire lesion, thereby improving detection of malignant transformation and optimizing biopsy site selection.

In this case, a patient with a rapidly evolving plantar macule underwent a punch biopsy with inconclusive results, followed by a shave biopsy consistent with lentigo. However, the clinical and dermoscopic features remained concerning for malignancy, prompting further evaluation with RCM. Imaging revealed a melanoma-characteristic pattern, guiding a single targeted rebiopsy that confirmed the diagnosis of ALM in situ. Absent confocal guidance, the next step in management would have required a broad incisional biopsy or multiple mapping biopsies. Both approaches have important limitations, including an association of the former with increased 5-year mortality in cutaneous melanoma and the inability of the latter to guarantee sampling of the most diagnostically relevant sites.^3^^,^^4^

Current studies support the value of RCM in identifying features of acral lesions that distinguish ALM from benign nevi, with a reported diagnostic accuracy of 90.3%, and show strong concordance with histopathology.^5^, ^6^, ^7^, ^8^ RCM likewise demonstrates high concordance with histopathology for presurgical margin mapping in lentigo maligna.^9^ Based on existing evidence and our experience, we recommend considering RCM as an adjunctive tool for precise biopsy site selection in diagnostically challenging acral lesions.

This case provided an opportunity to diagnose melanoma in a patient with high clinical suspicion despite nondiagnostic pathology, serving as an important reminder for dermatologists to continue pursuing their clinical judgment when discordant findings arise.

## Conflicts of interest

Dr Lilia Correa-Selm reports consulting relationships with Accutec Blades, Enspectra Health, and Novartis. She has served as a researcher for Novartis, Pfizer, and Sanofi. She has also participated as a speaker for La Roche-Posay. All other authors declare no conflicts of interest.
